# Case Report: When metabolism meets malabsorption: Imerslund-Gräsbeck syndrome with coexisting propionic acidemia presenting as recurrent metabolic crises

**DOI:** 10.3389/fnut.2026.1877112

**Published:** 2026-09-21

**Authors:** Anvitha Rallapalli, Shubham Raj, Sanjhi Paliwal, Vikas Bhatia, Savita Attri, Arushi Gahlot Saini

**Affiliations:** 1 Department of Pediatrics, Postgraduate Institute of Medical Education and Research, Chandigarh, India; 2 Department of Radiodiagnosis, Postgraduate Institute of Medical Education and Research, Chandigarh, India

**Keywords:** B12 deficiency, CUBILIN, CUBN, Imerslund-Gräsbeck syndrome, propionic acidemia

## Abstract

**Background:**

Propionic acidemia and Imerslund-Gräsbeck syndrome are rare inherited metabolic disorders with distinct pathophysiological mechanisms. Their occurrence in the same child is extremely unusual and can create diagnostic and therapeutic challenges because of overlapping neurological and hematological manifestations.

**Case:**

A 1.5-year-old boy presented with recurrent episodes of encephalopathy precipitated by febrile illnesses, associated with vomiting, severe metabolic acidosis, ketosis, and refractory focal status epilepticus. Following repeated metabolic crises, developmental stagnation became evident. On examination, he was encephalopathic with right-sided weakness, hypotonia, and brisk deep tendon reflexes. Investigations revealed high anion gap metabolic acidosis, mild hyperammonemia, neutropenia, microcytic anemia, and persistent proteinuria. Metabolic workup showed elevated propionyl carnitine with increased urinary 3-hydroxypropionic acid and propionyl glycine, suggestive of propionic acidemia. Whole-exome sequencing identified a homozygous pathogenic variant in the *PCCB* gene, confirming propionic acidemia, along with a novel homozygous stop-gain variant in the *CUBN* gene consistent with Imerslund-Gräsbeck syndrome. Neuroimaging demonstrated bilateral basal ganglia involvement, diffuse white matter abnormalities, and progressive cerebral atrophy, reflecting recurrent metabolic injury. The child was treated with anti-seizure medications, metabolic stabilization, protein-restricted diet, mitochondrial cocktail supplementation, and monthly vitamin B12 therapy.

**Conclusion:**

This case represents an exceptionally rare coexistence of propionic acidemia and Imerslund–Gräsbeck syndrome. It highlights the importance of considering dual pathology in children with atypical clinical or biochemical features and underscores the value of comprehensive genetic evaluation in complex neurometabolic disorders.

## Introduction

Imerslund-Gräsbeck syndrome is a rare, autosomal-recessive disorder characterized by selective intestinal malabsorption of cobalamin (vitamin B12). It is caused by biallelic pathogenic variations in two genes: CUBN gene encoding cubilin protein, and the AMN gene encoding amnionless protein. Both these proteins are components of the cubam-receptor complex responsible for cobalamin-intrinsic factor absorption in the terminal ileum. The cubam receptor is localized in the intestines and kidney, thus its deficiency leads to intestinal malabsorption of cobalamin and non-progressive proteinuria secondary to impaired proximal tubular reabsorption (1).

Propionic acidemia is another rare, autosomal recessive, inherited metabolic disorder caused by deficiency of propionyl-CoA carboxylase (PCC), a mitochondrial enzyme that catalyzes the conversion of propionyl-CoA to methylmalonyl-CoA (2). This enzyme deficiency results in accumulation of propionic acid and its metabolites, including 3-hydroxypropionic acid, methyl citric acid, and propionyl glycine, which are toxic to multiple organ systems (3). The genetic basis of PA involves mutations in two genes: PCCA (encoding the alpha subunit of PCC) and PCCB (encoding the beta subunit).

Despite affecting distinct biochemical pathways, Imerslund-Gräsbeck syndrome and propionic acidemia can have overlapping features such as anemia, neurological symptoms, and failure to thrive. Rarely, both these disorders may be seen in the same patients and may complicate the clinical picture and has implications in management. We discuss here an interesting case with the dual diagnosis of these two disorders which led to a personalized patient-care plan. Our case also highlights the importance of comprehensive genetic evaluation in children with atypical or evolving metabolic phenotypes.

## Clinical presentation

A 1.5-year-old boy presented with recurrent episodes of worsening encephalopathy following minor febrile illnesses from 1 year of age. He was first-born to non-consanguineous parents with a normal perinatal period. His developmental milestones were attained at appropriate age till 1 year of age. At 1.5 years of age, he presented to our center with fever, recurrent vomiting and encephalopathy for the past 1 week. On day 7 of illness, he had drug-refractory, right, focal, status epilepticus requiring mechanical ventilation.

## Past and family history

He had a similar episode of fever-triggered, progressive encephalopathy at 1 year of age which gradually improved with supportive care over the next 1 week. The family history was unremarkable.

## Examination

Anthropometric parameters at 1.5 years of age were preserved with weight 11.5 kg (1.4 z-score), length 82 cm (2.06 z-score), and head circumference 48 cm (1.38 z-score). There were no dysmorphic features, neurocutaneous markers, or abnormalities of skin and hair. At presentation, he was encephalopathic with tachycardia and acidotic breathing. Neurological examination revealed right hemiparesis with hypotonia and brisk deep tendon reflexes. Bilateral plantar responses were extensor. Cranial nerve examination was unremarkable. Other systemic examination findings were unremarkable.

## Hematological and biochemical investigations

Hemogram revealed microcytic hypochromic anemia with neutropenia: hemoglobin 83 g/L, mean corpuscular volume 73.5 fL, and absolute neutrophil count 0.955 × 10^9^/L. Arterial blood gas analysis showed severe high anion gap metabolic acidosis: pH 6.9, bicarbonate 1.8 mmol/L, pCO_2_ 7.71 mmHg, lactate 0.8 mmol/L, and anion gap 22 mmol/L. Blood ketones were elevated at 2.8 mmol/L. Serum ammonia was elevated at 91 μmol/L (institutional reference range: 17–68 μmol/L). Liver and renal function tests, serum electrolytes, and blood glucose were within normal limits. Urine protein-to-creatinine ratio was elevated at 2.5, suggesting proteinuria. Amino-acid and acyl carnitine profile by tandem mass spectrometry (TMS) demonstrated markedly elevated propionyl carnitine (C3) and C3/C2, C3/C16 ratios. The urine gas chromatogram by GCMS showed excretion of 3-hydroxypropionic acid and propionyl glycine (Figure 1). Together, these were suggestive of propionic acidemia. Following the initiation of metabolic stabilization and empirical vitamin therapy, post-supplementation laboratory evaluation revealed a serum vitamin B12 level of >2000 pg./mL and normal total homocysteine levels.

**Figure 1:**
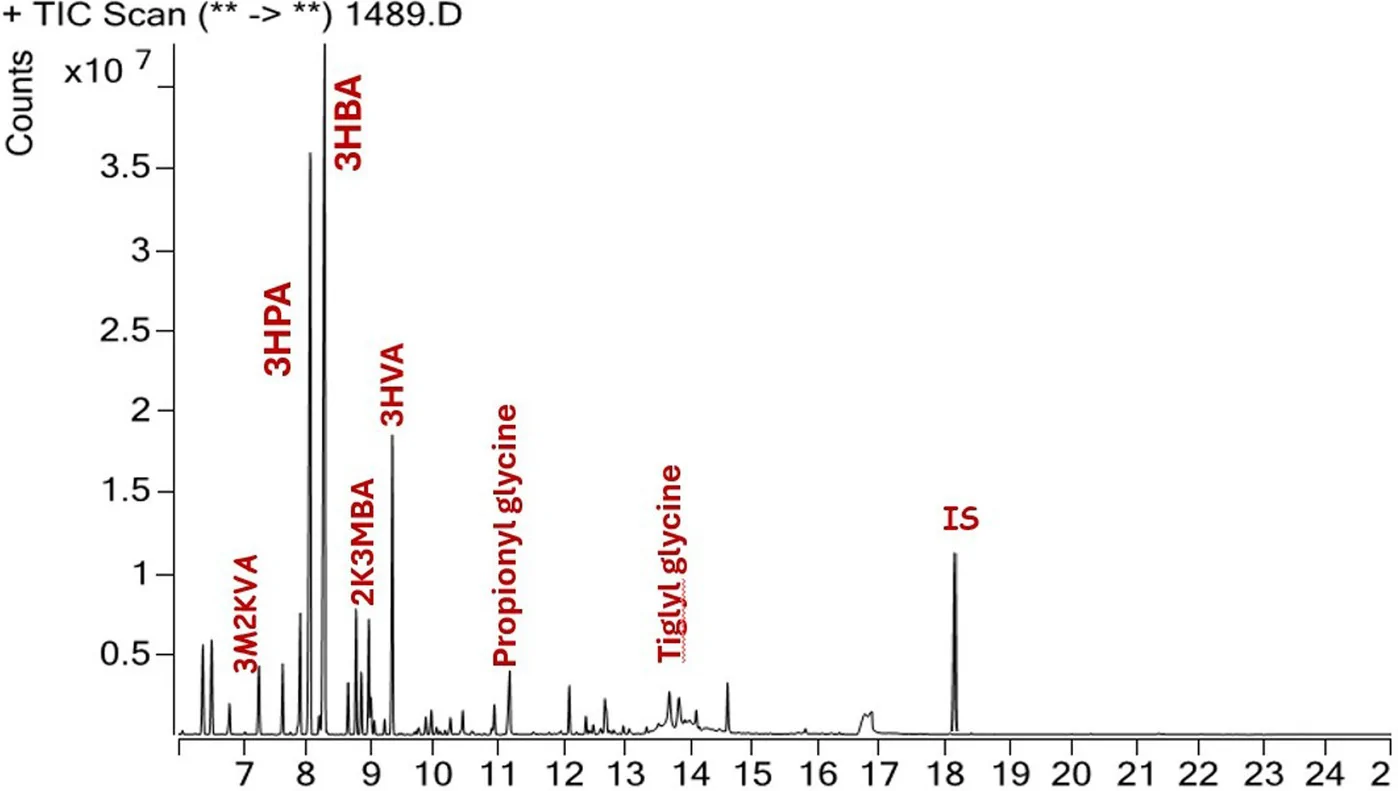
Total ion chromatogram by GCMS from the index patient with Propionic acidemia showing increased excretion of 3-hydroxypropionic acid and propionylglycine suggestive of Propionic acidemia. 3HPA = 3-Hydroxypropionic acid, 3HBA = 3-Hydroxybutyric acid, 3HVA = 3-Hydroxyvaleric acid, IS = Internal standard.

## Neuroimaging

Magnetic Resonance Imaging (MRI) of the brain at 1.5 year of age demonstrated symmetric areas of hyperintensity in bilateral globus pallidi, caudate, putamen and in both cerebral hemispheres. Notably, diffusion restriction was noted in bilateral globus pallidi. These hallmark radiological findings were highly suggestive of an underlying organic acidemia (Figure 2).

**Figure 2:**
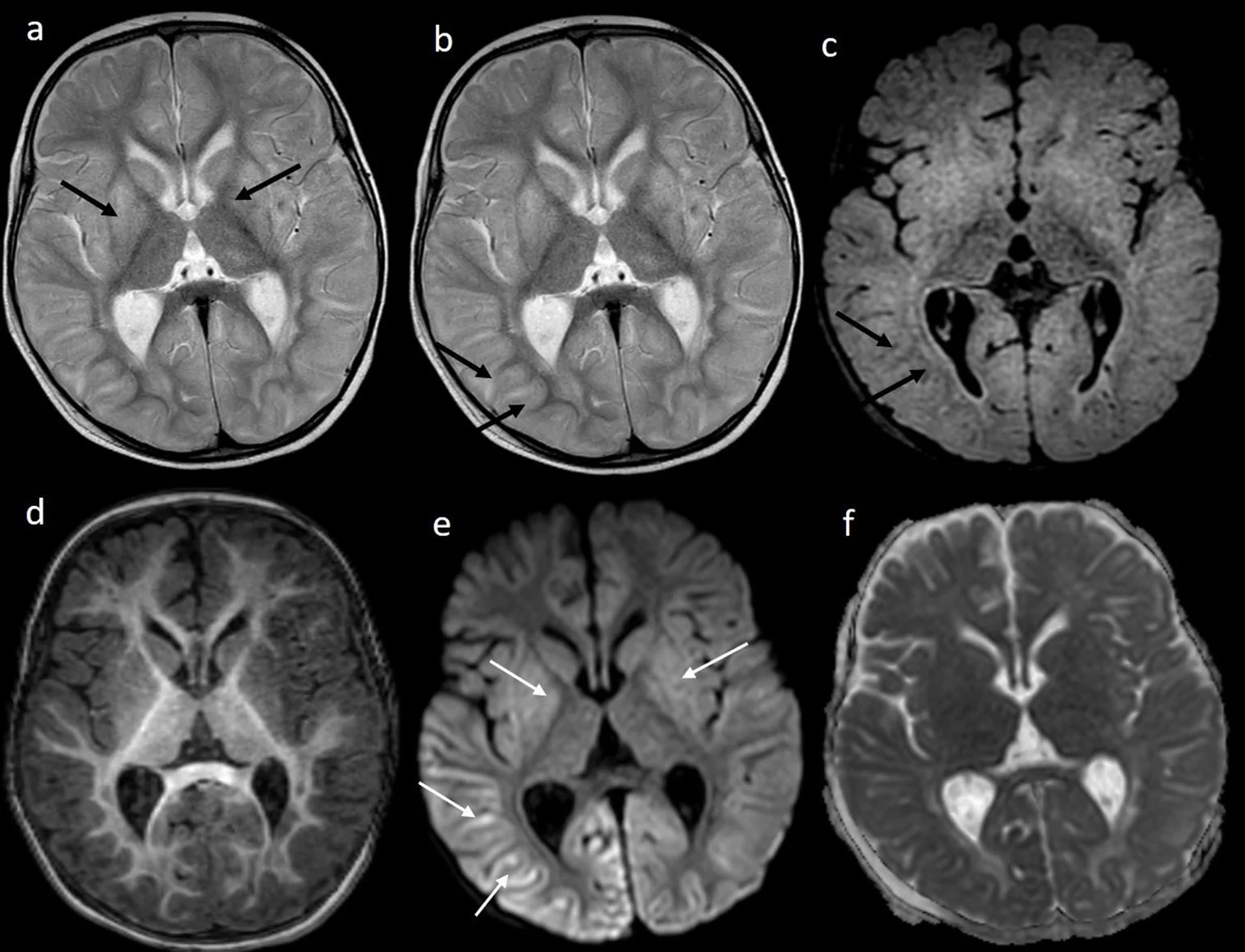
Magnetic resonance imaging at 1.5 years of age: axial T2-weighted **(a)** and FLAIR **(b)** images show hyperintensity (black arrows) involving the bilateral caudate, lentiform nuclei and subcortical occipital lobe white matter, which are hypointense on T1-weighted images **(c)**. Diffusion-weighted images **(d,e)** show intense diffusion restriction in globus pallidi, and the subcortical cerebral hemisphere regions (white arrows), which are hypointense on ADC image **(f)**.

## Genetic analysis

Peripheral blood sample was sent for whole-exome sequencing with Novaseq 6,000 platform to Centre for Cellular and Molecular Biology, Hyderabad. The Sentieon pipeline (v201808.01) was used to align reads to the human reference genome GRCh37/hg19. Sentieon’s Haplotyper module with duplicate removal, base recalibration, and indel realignment was used for small indels and single nucleotide variations through variant calling. ExomeDepth (v1.1.10) was used to identify copy number variants and standard databases (gnomAD, 1,000 Genomes, ClinVar) were used to annotate the variants. ACMG/AMP criteria were used for interpretation of variants (4). Sanger sequencing was used to analyze parental segregation. PolyPhen-2, SIFT, MutationTaster2, LRT, EVE, alpha missense and Meta RNN score were used to evaluate variant pathogenicity. An analysis of experimental 3D structure as well as the functional consequences of missense variant of the PCCB (Propionyl-CoA carboxylase beta chain) protein was conducted using HOPE software (Figure 3) (5). Whole exome sequencing identified a homozygous pathogenic variant in the *PCCB* gene, c.493C > T (p.Arg165Trp), confirming the diagnosis of Propionic Acidemia. An additional homozygous likely pathogenic variant in the *CUBN* gene, c.10339G > T (p.Glu3447Ter), was also detected, consistent with Imerslund-Grasbeck syndrome.

**Figure 3:**
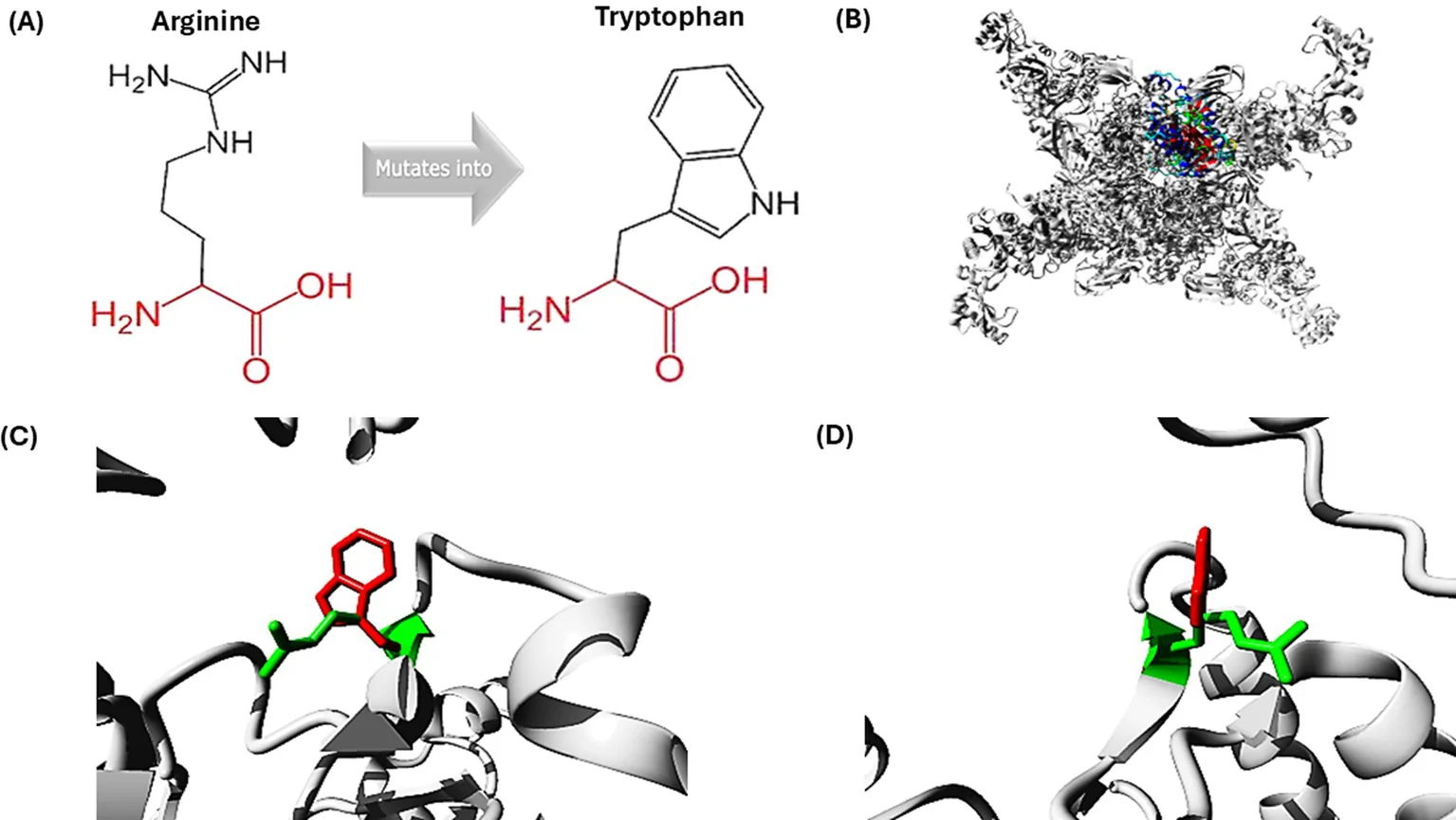
Three-dimensional PCCB protein structure for c.493C > T; p.Arg165Trp. **(A)** Schematic structures of the original and the mutant amino acid. The backbone is colored red. The side chain, unique for each amino acid, is colored black. **(B)** Overview of the protein in ribbon-presentation (*α*-helix = blue, *β*-strand = red, turn = green, 3/10helix = yellow and random coil = cyan and other molecules = grey). **(C,D)** Close-up of the mutation (protein = grey, the side chains of both the wild-type and the mutant residue are shown in green and red, respectively).

## Management

The child received sequential loading with multiple anti-seizure medications, including phenytoin, valproate, phenobarbitone, levetiracetam, and lacosamide; however, seizures persisted. In view of refractory seizures, midazolam infusion was started (up to 4 mcg/kg/min), after which seizures were controlled within 24 h. The infusion was gradually tapered and stopped by day 4 of hospitalization, with no further seizure recurrence.

In parallel, he was managed for metabolic crisis with intravenous dextrose-containing fluids, bicarbonate infusion and mitochondrial cocktail therapy including oral carnitine (300 mg/kg/day), Coenzyme Q (30 mg/day) and high dose vitamin supplementation. Over the following days, encephalopathy gradually improved, and he began to recognize his parents and accept oral feeds. To prevent metabolic catabolism and limit the propiogenic substrate burden, the child was started on a protein-restricted diet providing 0.8 g/kg/day of natural protein, with a total caloric intake maintained at approximately 100 kcal/kg/day. This diet was supplemented with a specialized, precursor-free metabolic formula tailored for propionic acidemia to meet age-appropriate protein and energy requirements.

## Outcome and follow-up

The child remains on regular longitudinal follow-up every 6 months in the neurometabolic clinic for comprehensive nutritional and biochemical surveillance. Monitoring parameters include detailed anthropometry alongside regular laboratory evaluations of serum albumin, prealbumin, complete blood count, liver and renal function tests, serum ammonia, urine ketones, and serum vitamin D levels, all of which have sustainably remained within normal parameters during follow-up. The child’s long-term maintenance regimen includes oral L-carnitine (300 mg/day), Coenzyme Q10 (100 mg/day), thiamine (100 mg/day), and riboflavin (200 mg/day). For gut decontamination, he receives cyclic oral metronidazole (10 mg/kg/day for 5 days each month), and for Imerslund-Gräsbeck syndrome, he receives monthly intramuscular vitamin b12 injections (1 mg) to bypass defective intestinal absorption. There are no clinical signs of B12 deficiency and his hemogram and indices remained normal. Following the diagnosis of Imerslund-Gräsbeck syndrome, the child was initiated on regular monthly intramuscular vitamin B12 injections. While subsequent follow-up hemograms and red cell indices remained entirely normal with no clinical signs of hematological cobalamin deficiency, this maintenance therapy did not prevent further metabolic decompensations. The child experienced two additional fever-triggered metabolic crises at ages 2 years and 2.5 years despite established compliance with B12 therapy. Each recurrent episode was characterized by encephalopathy, ketosis, and severe high anion gap metabolic acidosis requiring hospitalization and approximately 1 week for recovery. Following these recurrent metabolic crises, an accretion of deficits with developmental stagnation became evident. At his latest follow-up at 3 years of age, clinical assessment confirmed a severe global developmental delay; he could walk a few steps independently and possessed an expressive vocabulary of approximately 15 words, tracking at an approximate 15-month developmental age equivalent. His repeat neuroimaging showed similar areas of involvement (Figure 4).

**Figure 4:**
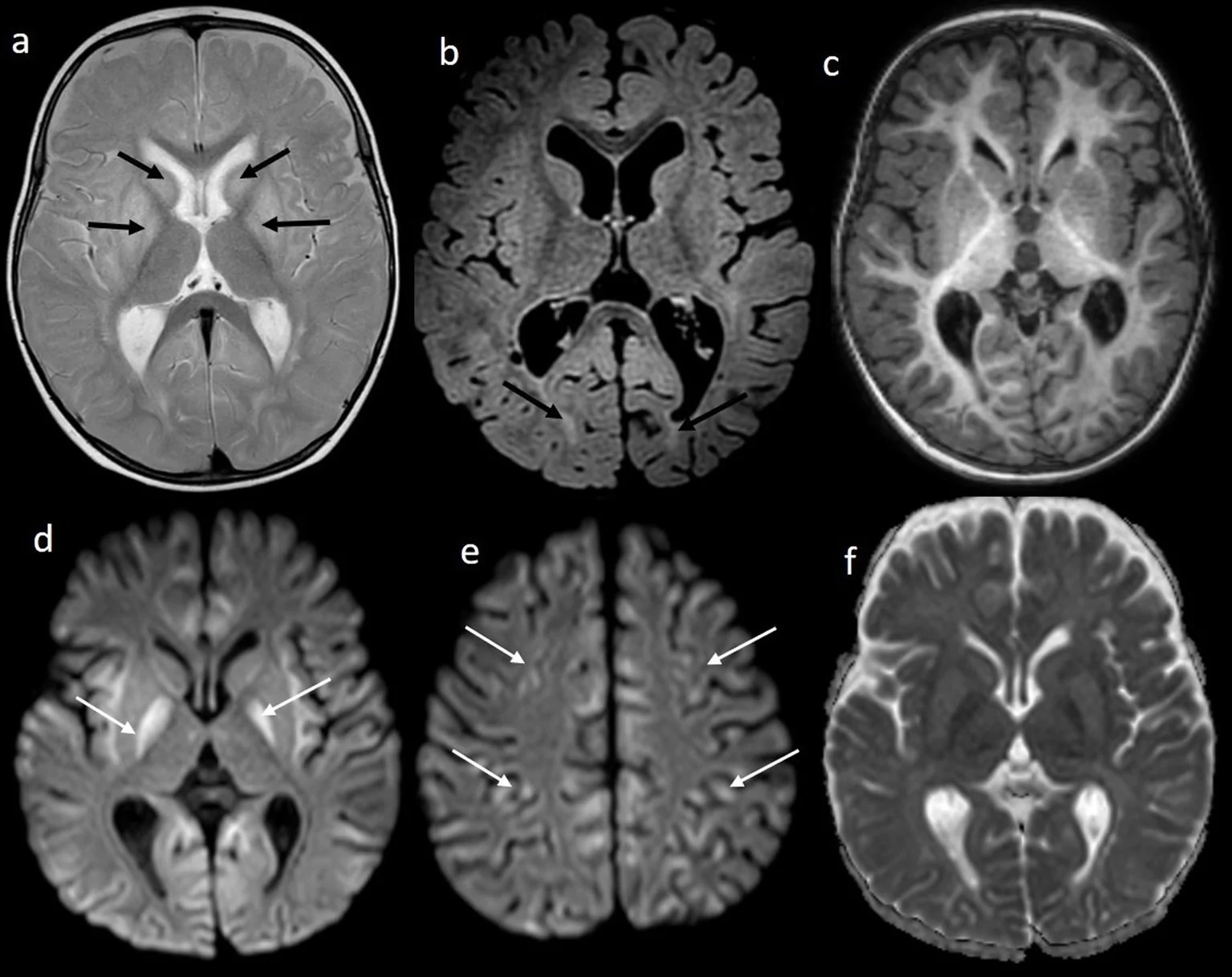
Magnetic resonance imaging at 2.5 years of age: Axial T2-weighted **(a,b)** and FLAIR **(c)** images show subtle hyperintensity (black arrows) involving the bilateral caudate, lentiform nuclei and cortical right occipital lobe, which are hypointense on T1-weighted images **(d)**. Diffusion-weighted images **(e)** show mild diffusion restriction in these regions (white arrows), which are hypointense on the ADC image **(f)**.

## Discussion

The coexistence of two individually rare inherited metabolic disorders, propionic acidemia and Imerslund-Gräsbeck syndrome, in the index child represents an unusual association. Propionic acidemia has global incidence generally estimated between 1 in 20,000 to 1 in 250,000 live births, though it varies significantly by region (6, 7). Incidence is higher in areas with higher rates of consanguinity, such as the Middle East, where it can be 1 in 2,000–5,000 live births. In USA, incidence is estimated to be 1 in 100,000–1,30,000 live births (8).

The most clinically relevant aspect of this case is the presence of a cobalamin transport defect in a child with established organic acidemia. Although vitamin B12 deficiency is classically associated with megaloblastic anemia and hyperhomocysteinemia, disorders of intracellular transport and receptor-mediated absorption may present with atypical biochemical profiles (9). The coexistence of Imerslund-Gräsbeck syndrome compromises metabolic resilience in patients with underlying propionic acidemia. Vitamin B12 is an essential cofactor for methyl malonyl-CoA mutase, a critical mitochondrial enzyme downstream of propionyl-CoA carboxylase (PCC). When Imerslund-Gräsbeck syndrome causes a functional intracellular cobalamin deficiency, it effectively creates a secondary metabolic bottleneck immediately following the primary PCC block. This dual enzymatic impairment drastically compromises ATP production and mitochondrial intermediary metabolism. Consequently, during periods of intercurrent febrile illness, the patient’s capacity to tolerate catabolic stress is profoundly diminished, precipitating severe high anion gap metabolic acidosis and recurrent encephalopathy (3). Crucially, clinicians must note that a normal or paradoxically elevated serum vitamin B12 level-as observed in our patient (due to prior B12 supplementation)- does not rule out functional tissue deficiency in IGS, as passive non-cubilin-dependent absorption pathways or dietary intake can artificially elevate circulating levels while intracellular starvation persists. However, because the patient’s subsequent metabolic crises at 2 and 2.5 years of age occurred after the implementation of regular intramuscular vitamin B12 therapy, the clinical course suggests that these acute decompensations were predominantly driven by the severe upstream enzymatic block of propionic acidemia. While a secondary mitochondrial bottleneck from intracellular cobalamin deficiency remains biologically plausible, its definitive contribution to the frequency of metabolic crises cannot be formally decoupled from the primary organic acidemia phenotype in this patient. Although both disorders independently affect intermediary metabolism and may share overlapping manifestations such as developmental delay, failure to thrive, neurological dysfunction, and hematological abnormalities, their simultaneous occurrence has not been well described in literature. Published reports involving Imerslund-Gräsbeck syndrome largely describe isolated vitamin B12 malabsorption, occasionally associated with elevated methylmalonic acid levels mimicking an inherited metabolic disorder. This case also underscores the importance of precision nutritional therapy in inherited metabolic diseases. Children with propionic acidemia require long-term protein restriction to reduce propiogenic substrate burden; however, excessive restriction risks micronutrient deficiency, growth failure, and impaired neurodevelopment (10, 11). The coexistence of a cobalamin absorption disorder further complicates nutritional planning and necessitates individualized supplementation strategies. Integrated metabolic and nutritional monitoring is therefore critical to optimize long-term outcomes.

The genetic findings in our patient further strengthen the uniqueness of this association. The child harbored a known homozygous pathogenic variant, c.493C > T; p.Arg165Trp, in exon 5 of the *PCCB* gene encoding the beta subunit of propionyl-CoA carboxylase. This variant lies within the ‘carboxyltransferase N-terminal’ region of the protein and has been previously associated with propionic acidemia. Structural modeling suggests that substitution of arginine by tryptophan alters the physicochemical properties of the residue, producing a bulkier and more hydrophobic amino acid within the protein core. This disrupts important hydrogen bonding and ionic interactions involving Asp105, Ser132, and Glu168, thereby affecting protein stability and enzymatic function (Figure 3). Based on ACMG classification, the variant fulfills multiple pathogenicity criteria including PS3, PS4, PM1, PM2, PM5, PP3, PP4, and PP5, and is categorized as pathogenic (12–15). In addition, the patient was found to harbor a novel homozygous stop-gain variant, c.10339G > T; p.Glu3447Ter, in exon 64 of the *CUBN* gene encoding cubilin, consistent with Imerslund–Gräsbeck syndrome. This nonsense mutation introduces a premature termination codon that is predicted either to generate a truncated nonfunctional protein or lead to nonsense-mediated mRNA decay (12). Notably, the variant was absent from major population databases including 1,000 Genomes, EVS, gnomAD, and the Indian Exome Database, and had not been previously reported in OMIM, HGMD, or published literature related to *CUBN* mutations. However, the variant has been submitted to ClinVar (Accession: VCV003774533.1) and was consistently predicted to be deleterious by multiple in-silico prediction tools including MutationTaster2021, Franklin, VarSome, and InterVar. According to ACMG criteria, the variant satisfies PVS1, PM2, and PP3, supporting its classification as pathogenic. The simultaneous presence of pathogenic variants in both *PCCB* and *CUBN* genes highlights the importance of comprehensive genomic evaluation in children with atypical or multisystem metabolic phenotypes, particularly in populations with high rates of consanguinity.

MRI findings in our patient evolved with age and recurrent metabolic decompensation. During the crisis at 18 months of age, MRI demonstrated symmetric hyperintensities with diffusion restriction involving the bilateral caudate nuclei, putamina, and globi pallidi, along with diffuse cortical–subcortical white matter abnormalities, consistent with acute metabolic encephalopathy. Follow-up MRI at 30 months of age showed interval progression with diffuse cerebral atrophy, thinning of the corpus callosum, persistent bilateral white matter signal abnormalities, and new cortical diffusion restriction involving the right parieto-temporo-occipital and frontal cortices, suggesting recurrent metabolic injury and chronic neurodegenerative sequelae. Basal ganglia involvement, especially affecting the striatum including the putamen and caudate nuclei, is considered a characteristic neuroimaging abnormality in propionic acidemia and reflects the selective vulnerability of metabolically active deep gray matter structures during metabolic decompensation (16). Neuroimaging patterns in propionic acidemia are known to vary with age; neonates typically demonstrate diffuse edema and signal abnormalities in the cerebral white matter with relative sparing of the basal ganglia, whereas older children more commonly develop hyperintense lesions within the basal ganglia, particularly involving the putamen and caudate nuclei (17). Abnormal signal intensity and edema within the cerebral and cerebellar cortices have also been described, although less frequently. Diffusion-weighted imaging in both neonates and older children characteristically demonstrates restricted diffusion in the affected regions during episodes of metabolic decompensation (18). During the chronic untreated form of the disease, abnormal myelination as well as cortical and white matter atrophy can be expected, findings that parallel the progressive atrophic and white matter changes observed in our patient (18).

In conclusion, this case expands the clinical spectrum of both propionic acidemia and Imerslund-Gräsbeck syndrome, and highlights the importance of considering dual pathology in children with atypical neurometabolic presentations. Normal serum B12 levels do not exclude clinically significant defects in cobalamin pathways. Comprehensive metabolic testing, early genetic evaluation and integrated nutritional-metabolic management are essential to prevent recurrent crises and progressive neurological damage in children with complex inherited metabolic disorders.

## Data Availability

The original contributions presented in the study are included in the article/supplementary material, further inquiries can be directed to the corresponding authors.
